# What have we learned from a decade treating patients with diabetic macular oedema with 0.19 mg fluocinolone acetonide intravitreal implant?

**DOI:** 10.1038/s41433-025-03692-7

**Published:** 2025-02-19

**Authors:** Laurent Kodjikian, Lilianne Duarte, Pankaj Singh, Maged Habib, Victor Gonzalez

**Affiliations:** 1 Service d’Ophtalmologie, Hôpital Universitaire de la Croix-Rousse, Hospices Civils de Lyon, Lyon, France; 2 UMR5510 MATEIS, CNRS, INSA Lyon, Université Lyon 1, Villeurbanne, France; 3 Department of Ophthalmology, Centro Hospitalar de Entre O Douro E Vouga, Santa Maria da Feira, Portugal; 4 Department of Ophthalmology, Goethe University Hospital, Frankfurt am Main, Germany; 5 Department of Ophthalmology, Institute of Eye Surgery. Waterford. Ireland, Waterford, Ireland; 6 Valley Retina Institute, McAllen, TX USA

**Keywords:** Retinal diseases, Vision disorders

## Abstract

Diabetic macular oedema [DMO] is a prevalent and sight-threatening condition among diabetic patients, which can cause irreversible blindness. Since angiogenesis and inflammation are two key elements in the etiopathogenesis of DMO, intravitreal injections of vascular endothelial growth factor inhibitors [anti-VEGF] and sustained released intravitreal corticosteroid implants are currently considered as treatments of choice. The introduction, 10 years ago, of the 0.19 mg fluocinolone acetonide [FAc] implant for treating eyes with vision impairment associated with recurrent and persistent DMO represented an important advance. Since then, two randomized-control trials and many real-world studies have shown its good efficacy/safety profile and the replicability of its treatment regimen. The FAc implant is, in general terms well tolerated, although it is associated with intraocular pressure-[IOP] and cataract-related adverse events [AEs]. Most IOP-related AEs are effectively controlled with ocular-hypotensive therapies. The objective of this paper is to review the role of FAc implant in the treatment of DMO over the 10 years since its launch, as well as its impact on clinical practice outcomes.

## Introduction

The prevalence of diabetes mellitus [DM] and diabetes related diseases have significantly increased over the last decades worldwide, mainly due to aging of the population and the current lifestyle [1–3]. Therefore, DM entails a significant burden on health services [4, 5].

Amongst patients with DM, Diabetic Retinopathy [DR] and Diabetic Macular Oedema [DMO] are the most common complications leading to vision loss [3]. It was estimated that 103.1 million, 28.5 million, and 18.8 million people worldwide were diagnosed with DR, vision-threatening DR, and DMO, respectively, in 2020 [4]; with a likely increase in 2030 to 129.8 million for DR, 44.8 million for vision-threatening DR and 23.5 million for DMO [4]. In fact, in Europe, the incidence per year of DMO in patients with type-2-DM was 0.4%, with a prevalence of 3.7% [2].

DR/DMO are multifactorial diseases and involve different factors in their pathological process [6–11]. Among them, inflammation and angiogenesis can be triggered by the same molecular events, in which ischemia plays an important role, further strengthening this association [12, 13].

There are different options for treating patients with DMO, including, intravitreal corticosteroids and inhibitors of the vascular endothelial growth factor [anti-VEGF], the latter are considered first-line treatments [14]. In recent years, intravitreal injections [either anti-VEGF or corticosteroids therapies] have become the standard of care for centre involve DMO treatment [14–17].

The 0.19 mg fluocinolone acetonide [FAc] implant was approved in several European countries for treating eyes with vision impairment associated with recurrent (i.e., periodic DMO episodes that recur after initial resolution) and recalcitrant DMO (i.e., a more severe form of DMO that is resistant to conventional intravitreal treatments) [18, 19]. Additionally, the 0.19 mg FAc implant is also indicated for the prevention of relapse in recurrent non-infectious uveitis affecting the posterior segment of the eye [19].

Different studies, either from randomised control trials [20–22] or from real-world practice [23–44] have evaluated the effectiveness and safety of the 0.19 mg FAc implant throughout the last 10 years. Overall, they all point in the same direction, demonstrating the good efficacy and safety profile, as well as the replicability of treatment regimen of the 0.19 mg FAc implant. Indeed, the results of a review paper that included data from 22 studies conducted in a real-life scenario, reported a mean visual improvement of +8.7 letters [range: 0.4 to 18.8 letters, median +8.0 letters] and a maximum central retinal thickness [CRT] reduction of −34.3% [range: −10.7 to −55.8%, median: −36.2%] from baseline [34].

It has been 10 years since the launch of FAc implant, the first long-lasting corticosteroid intravitreal implant for the treatment of DMO [18, 19]. Its introduction represented an important change in the therapeutic management of DMO. In these 10 years, FAc implant has become a valuable option for treating eyes with vision impairment associated with chronic recurrent and recalcitrant DMO who did not respond sufficiently to other available therapies [20–44].

This paper aims to review the impact that the 0.19 mg fluocinolone acetonide intravitreal implant has had on the clinical management and the treatment paradigm of patients with DMO over these 10 years.

This paper will be focus on evaluating different aspects related to the FAc implant, including the evidence about its use in randomised clinical trials and routine clinical practice, the incidence and types of adverse events, and some specific situations, such as, the role of the FAc implant in the current DMO treatment paradigm.

## Methods

This was a comprehensive narrative review of the currently available evidence evaluating the efficacy and safety of the 0.19 mg FAc implant in patients with DMO.

### Search strategy and summary of eligibility criteria, outcomes, and data collection

#### Search strategy

A comprehensive but not systematic search of PubMed, Medline, Embase, and Google Scholar databases was conducted to identify randomised controlled trials (RCTs) and real-world evidence studies assessing the efficacy and safety of the 0.19 mg fluocinolone acetonide (FAc) implant in patients with DMO between January 2011 and May 2024.

The search strategy utilised Medical Subject Headings (MeSH) terms, including “Fluocinolone acetonide implant” AND “DMO.” Additionally, a free-text search of titles and abstracts was performed using relevant keywords, such as “DMO” OR “DMO/DME” AND “Fluocinolone acetonide implant” OR “ILUVIEN”.

This structured approach ensured rigorous inclusion and high-quality data for evaluated the FAc implant and its clinical implications in patients with DMO.

#### Eligibility criteria

- Inclusion: Studies using 0.2 µg/day FAc intravitreal implant for chronic DMO, reporting outcomes at ≥24 months, with a minimum of 10 patients, and data on the primary outcome.
- Exclusion: Abstracts; conference posters; reports on only vitrectomized eyes; non-peer-reviewed; studies published in other language different from English, French, Portuguese, Italian, or Spanish; or conditions other than DME were excluded.
- Study Design: No restrictions; real-world data outside RCTs were included.

#### Outcomes

- Mean change in BCVA at 24 months.
- BCVA change at 36 months.
- Change in OCT-measured CMT.
- Rates of supplementary intravitreal therapy, cataract surgery, IOP-lowering drops, and glaucoma surgery.
- CMT defined as the fovea-centred average within 1 mm diameter.
- Supplementary therapy excluded repeated FAc implants.

#### Data collection

- Data Extraction: Included author, year, study design/location, patient demographics, follow-up duration, BCVA/CMT changes, and rates of additional therapies and surgeries.

## Pharmacokinetics of 0.19 mg Fluocinolone Acetonide Intravitreal Implant

The 0.19 mg FAc implant [ILUVIEN^®^; Alimera Sciences, Dublin, Ireland] is a nonbiodegradable insert implanted in the vitreous via injection through the pars plana using a 25-gauge needle, which contains 0.19 mg of fluocinolone acetonide [19]. After a rapid increase in the concentration of fluocinolone acetonide, which reaches its peak approximately one week after implantation, there is a slight decrease in the concentration from first 3–-6 months, which is followed by a stable, continuous, and sustained delivery of 0.2 µg/day of fluocinolone acetonide through to month 36 [18].

## Effectiveness of 0.19 mg fluocinolone acetonide intravitreal implant

### Randomised control trials

Two RCTs have evaluated the effectiveness and safety of two FAc implants, i.e., 0.2 μg/day [low-dose] or 0.5 μg/day [high-dose] in patients with DMO [44, 45].

The FAME trials consisted of two pivotal RCT, sham-injection controlled, that compared the effectiveness and safety of two FAc implants [0.2 μg/day [low-dose] or 0.5 μg/day [high-dose]] versus sham injection in patients with DMO [20].

At month 24, the proportion of patients achieving a best-corrected visual acuity (BCVA) improvement ≥15 letters was significantly greater in both low-and high-dose groups, when compared to the sham group [*p* = 0.002 both]. Mean BCVA improvement was 4.4, 5.4, and 1.7 in the low-dose, high-dose, and sham, respectively [low-dose vs sham, *p* = 0.02 and high-dose vs sham *p* = 0.016]. Comparing with the sham group, the central retinal thickness [CRT] reduction was significantly greater in both low- and high-dose at all the time points measured [20].

At month 36, the proportion of eyes who achieved a BCVA improvement ≥15 ETDRS letters was 28.7% [*p* = 0.018 vs Sham], 27.8%, and 18.9% in the 0.2 μg/day, 0.5 μg/day, and sham groups, respectively [44]. In a subgroup analysis that included eyes with chronic DMO (chronic DMO as lasting more than 3 years) [44], the percentage of eye who achieved a gain in BCVA ≥ 15 ETDRS letters was significantly greater in the FAc implants groups when compared with sham [FAc 0.2 μg/day, 34.0% vs. Sham, 13.4%; *p* < 0.001] and [FAc 0.5 μg/day, 28.8.0% vs. Sham, 13.4%; *p* = 0.002]. Comparing to baseline CRT was significantly reduced in the FAc 0.2 μg/day group.

Additionally, eyes with chronic DMO and nonchronic [duration from diagnosis, <3 years] were compared [22]. At month 36, proportion of patients gaining ≥15 letters in BCVA was significantly greater in patients with chronic DMO [FAc 0.2 μg/day, 34.0% vs. Sham, 13.4%; *p* < 0.001], but not in those with nonchronic DMO [FAc 0.2 μg/day, 22.3% vs. sham, 27.8%; *p* = 0.275].

A post hoc analysis of the FAME trials evaluated the treatment outcomes in phakic eyes who received the FAc 0.2 μg/day implant [44]. At month 36, the proportion of eyes achieving a BCVA improvement ≥15 letters was slightly higher in the eyes who had cataract surgery after [35.1%] than in the eyes who had cataract surgery before [29.3%]. Additionally, as compared with nonchronic DMO [27.5%], a greater proportion of eyes with chronic DMO [42.3%] achieved a BCVA improvement ≥15-letter [44].

The Table S1 summarises the main findings of the FAME trials.

### Real-World Studies

Since the 0.19 mg FAc implant was approved in Europe in 2012 for treating eyes with chronic DMO, who were considered insufficiently responsive to available therapies [19], many observational studies have assessed the implants’ effectiveness and safety in a clinical setting [23–43].

Regarding functional results in real-life studies [38], they are similar to those obtained in the FAME studies [20, 21]. A meta-analysis, comparing real-world data regarding the 0.19 mg FAc implant for chronic DMO with those results of the FAME trials was performed [38]. The primary outcome assessed was the mean change in BCVA at 24 months. Secondary outcomes included the 36-month mean BCVA, changes in central macular thickness (CMT), the incidence of supplementary intravitreal therapy, cataract surgery, use of intraocular pressure (IOP)-lowering medications, and glaucoma surgery [38]. A total of 9 real-world studies (7 retrospective and 2 prospective) were included in this systematic review. The results of this meta-analysis found a visual acuity improvement of +4.52 and +7.89 letters at 24 and 36 months, respectively [38]. These numbers agree with those described in the FAME studies [+4.4 letters at month 24 and +8.1 letters at month 36] [20, 21].

Table 1 summarises the main clinical outcomes of different real-life studies published in the last decade.

**Table 1:** Summary of real-world fluocinolone acetonide implant studies.

Study	Ref	Eyes (n)	LOFU, months	Mean BCVA difference (letters or logMAR) between baseline and at end of the follow-up	Mean CRT (μm) change from baseline at end of follow-up	Maximum BCVA change (letters or logMAR)	Maximum CRT change (μm)
Alfaqawi et al.	23	28	12	+8.0 letters	−40.1%	+8.0 letters	−40.1%
MEDISOFT	24	341	14	+5.3 letters	−21.2%	+5.3 letters	−21.2%
Fusi-Rubiano et al	25	29	36	+11.0 letters	−114	+11.0 letters	−124 μ
USER	26	160	24	N.A.	−25.3%	N.A.	−25.3%
Young et al	27	21	36	+9.3 letters	−157.8	+13.1 letters	−172.8
Retro-IDEAL	28	81	36	+2.7 letters	−158.0	+5.5 letters	−225.0
PALADIN	30	115	24	Baseline BCVA ≥ 20/40: +1.0 Baseline BCVA < 20/40: +3.0	−71.4	Baseline BCVA ≥ 20/40: +1.0 Baseline BCVA < 20/40: +6.1	−83.3
Panos et al	31	24	36	−0.15 logMAR	−132	−0.15 logMAR	−132
Mushtaq et al	32	96	36	+4.0 letters	−266.9	+5.5 letters	−266.9 μ
MEDISOFT	33	256	36	+4.5 letters	−26.0%*	+5.0 letters	−26.0%*
REALFAc	35	62	24	+4.8 letters	−27.6	+5.0 ± 12.7 letters	−52.8
PALADIN	36	94	36	+3.61 letters	−59.0 ± 123.6	+3.71 letters	N.A.
Elbarky et al	37	22	12	+25.5 ± 13.0 letters	−246.2 ± 93.4	+25.5 ± 13.0 letters	−246.2 ± 93.4
Khoramnia et al	39	695	24 36	+4.4 (1.8–7.0)^a^ letters +4.9 (2.1–7.7)^a^ letters	N.A.	+4.9 (2.1–7.7)^a^ letters	N.A.
Merrill et al	40	202	36	+4.5 letters	N.A.	N.A.	N.A.
Ruiz-Moreno et al	42	31	24	+6.3 (−0.0 to 14.6)^a^ +8.3 ± 14.0^c^	−140.6 (−218.6 to 62.6)^a^	+6.3 (−0.0 to 14.6)^a^ +8.3 ± 14.0^c^	−140.6 (−218.6 to 62.6)
Capone et al	46	241	24	+ 5.1 (2.6 to 7.5)^a^	−189 (−151 to −227)^a^	+5.6 (3.2 to 7.9)^a^	−191 (−155 to −227)
Review papers	Ref	Eyes (n)	LOFU, months	Mean BCVA difference (letters or logMAR) between baseline and at end of the follow-up	Mean CRT (μm) change from baseline at end of follow-up	Maximum BCVA change (letters or logMAR)	Maximum CRT change (μm)
Kodjikian et al**	34	N.A.	20^⁑^	+8.7 letters	−34.3%	+8.7 letters	−34.3%
Fallico et al***	38	428	24 36	+4.52 (2.56–6.48)^a^ letters +8.10 (6.34–9.86)^a^ letters	−167.8 (−193.3 to −143.3)^a^ −180.8 (−205.9 to 175.7)^a^	N.A.	N.A.

*Ref* Reference, *LOFU* Length of follow-up, *BCVA* Best corrected visual acuity, *CRT* Central retinal thickness, *NA* Not available, *ETDRS* Early Treatment Diabetic Retinopathy Study.

^*^Only 66 patients have recorded central retinal thickness measurements.

**Review that included data from 22 real-life studies.

***Systematic review and meta-analysis.

^⁑^Range 8.5 to 36 months, median 18.0 months.

^a^95% Confidence interval.

^b^Overall study population (*p* = 0.0510).

^c^Eyes with a baseline BCVA ≤ 70 ETDRS letters (*p* = 0.0165).

The Retro-IDEAL study was a retrospective and multicentre study conducted on 16 centres in Germany [28]. It included 81 eyes with persistent or recurrent DMO who did not adequately respond to a first-line intravitreal therapy. Comparing to baseline, the mean CRT was significantly reduced at all the time-points [28]. At month 36, BCVA gain was +2.7 letters, with a maximum BCVA change of +5.5 letters at month 18 and +5.4 letters at month-30 [28].

An audit of data from patients with chronic DMO treated with the 0.19 mg FAc implant across 14 centres in the United Kingdom was performed [33]. This study included 256 eyes with chronic DMO with a minimum of 3 years of follow-up [mean follow-up duration of 4.28 years]. Mean best reported visual acuity [BRVA] increased from 52.6 letters at baseline to 56.7 letters at month 3 and remained stable or the follow-up period of ≥3 years; with 44.4%, 30.9%, and 19.1% eyes with ≥5, ≥10, and ≥15 letters gained at month-36, respectively [33].

Regarding anatomic outcomes, retinal thickness was evaluated in 66 eyes with a reduction of central foveal thickness from 460.3 μm at baseline to 368.5 μm one month after FAc implant injection and to 340.5 μm at the at month-36 visit post-FAc implant injection [33].

**Fig. 1:**
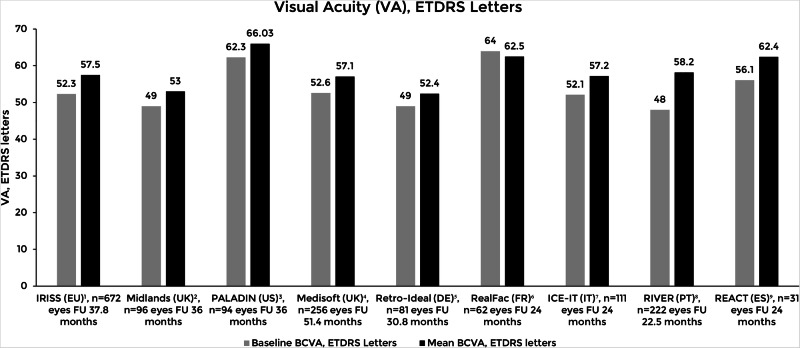
Overview of the mean visual acuity at baseline and the last follow-up visit in different real-world studies. Adapted from Augustin et al. [28]; Mushtaq et al. [32]; Bailey et al. [33]; Mathis et al. [35]; Khoramnia et al. [39]; Singer et al. [36]; Ruiz-Moreno et al. [42]; Capone et al. [46]; and Teixeira et al. [48]. Studies: IRIS [39]; Midlands [32]; PALADIN [36]; Medisoft [33]; Retro-IDEAL [28]; RealFac [35]; ICE-IT [46]; RIVER [48]; REACT [42]. BCVA: Best corrected visual acuity; ETDRS: Early Treatment Diabetic Retinopathy Study.

**Fig. 2:**
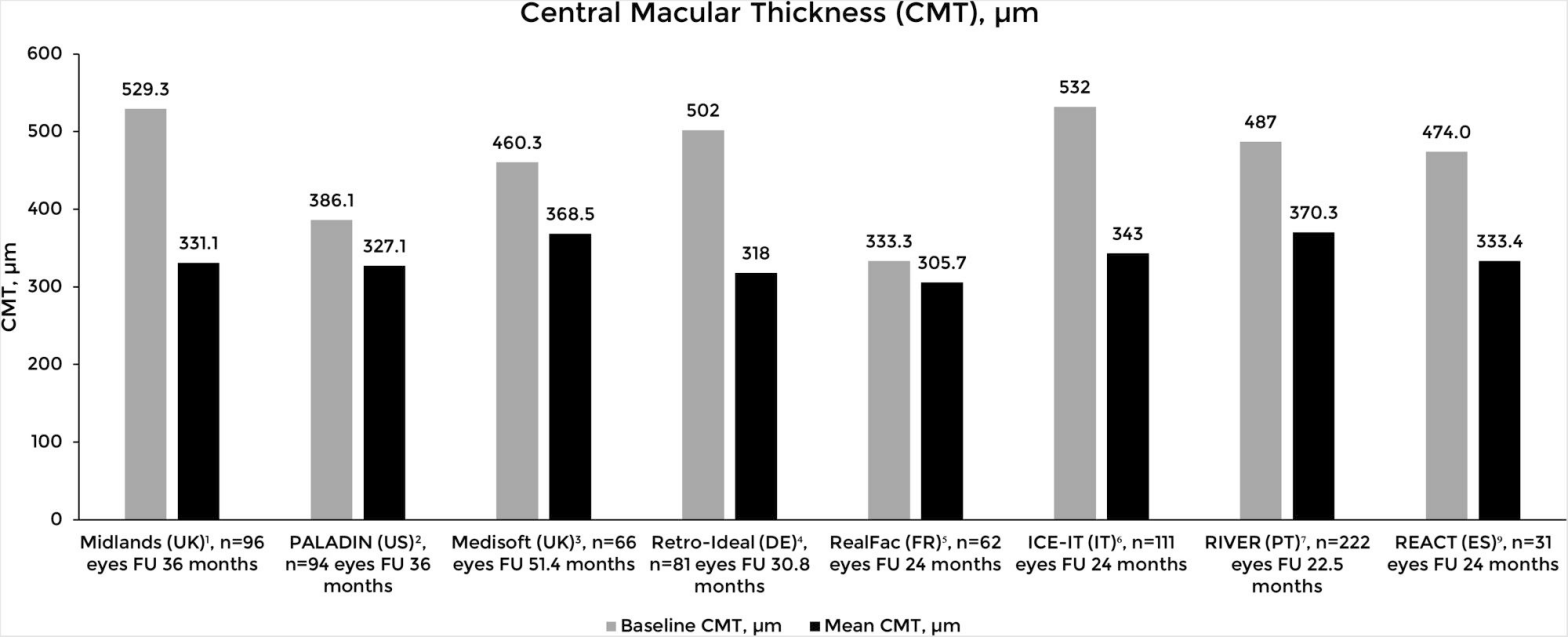
Overview of the mean central macular thickness at baseline and the last follow-up visit in different real-world studies. Adapted from Augustin et al. [28]; Mushtaq et al. [32]; Bailey et al. [33]; Mathis et al. [35]; Singer et al. [36]; Ruiz-Moreno et al. [42]; Capone et al. [46]; and Teixeira et al. [48]. Studies: Midlands [32]; PALADIN [36]; Medisoft [33]; Retro-IDEAL [28]; RealFac [35]; ICE-IT[46]; RIVER [48]; REACT [42]. CMT: Central macular thickness.

An overview of the main long-term follow-up real-world studies visual and anatomic outcomes are shown in Figs. 1 and 2, respectively.

## Safety of 0.19 mg fluocinolone acetonide intravitreal implant

### Randomised control trials

The most reported primary adverse events were increase of IOP and cataract [21, 22, 44, 45].

In the FAME studies, cataract extraction occurred in 80.0%, 87.2%, and 27.3% in the 0.2 μg/day FAc implant, 0.5 μg/day FAc implant and the sham group, respectively. Regarding the increase in IOP, it occurred in 37.1%, 45.5%, and 11.9% of eyes in the 0.2 μg/day FAc implant, 0.5 μg/day FAc implant and the sham group, respectively [21].

In the chronic DMO subgroup analysis, 85.1% of eyes from the 0.2 μg/day FAc implant group and 36.4% from the sham group required cataract surgery. In this chronic population, the need for IOP-lowering medication was 35.9% and 15.2% in the 0.2 μg/day FAc implant group and sham group, respectively [22].

Table S2 shows the rate of cataract related adverse events [AEs], cataract surgery, and IOP related AEs in the FAME trials.

### Real-world studies

Similarly to the RCTs, the most common AEs drug-related were IOP increase and cataract.

According to the results of a meta-analysis that included 22 observational real-world studies [1880 eyes], 20.1% of patients had a FAc-induced ocular hypertension [OHT] during the follow-up period. IOP-lowering medication was needed in 23.4% of patients and only 0.6% of patients needed IOP-lowering surgery [34].

Fallico et al [38], in a systematic review with meta-analysis of real-world studies, reported that 27% of eyes started IOP lowering medication during the study and 3% required any type of incisional IOP-lowering surgery.

**Fig. 3:**
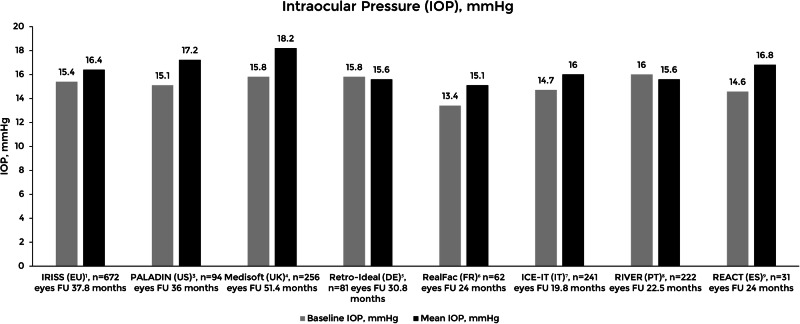
Overview of the mean intraocular pressure (IOP) at baseline and the last follow-up visit in different real-world studies. Adapted from Augustin et al. [28]; Mushtaq et al. [32]; Bailey et al. [33]; Mathis et al. [35]; Singer et al. [36]; Ruiz-Moreno et al. [42]; Capone et al. [46]; and Teixeira et al. [48]. Studies: Midlands [32]; PALADIN [36]; Medisoft [33]; Retro-IDEAL [28]; RealFac [35]; ICE-IT[46]; RIVER [48]; REACT [42]. IOP: Intraocular pressure.

Figure 3 shows the mean IOP at baseline and at the end of the study.

Figure S1 shows the proportion of emergent IOP-lowering medications and surgical/non-surgical procedures throughout the follow-up.

Regarding cataract related AEs, amongst phakic eyes, 43.2% required cataract surgery, with a mean time between FAc implant injection and cataract surgery of 8.1 months [34]. This data was similar to those reported by Fallico et al. [38], who found that 39% of eyes underwent cataract surgery.

The cataract related AEs and IOP related AEs reported by the different real-world studies have been reported in the Table S3.

## How has FAc implant changed the treatment paradigm in patients with DMO?

So far, we have reviewed the evidence that supported the use of the 0.19 mg FAc implant in patients with DMO, both in RCT and real-life clinical settings. The results from the real-life clinical studies demonstrated the 0.19 mg FAc implant provided a substantial visual benefit for up to 3 years in a much larger and heterogeneous population with DMO when compared to the population included in the FAME trials [44, 45].

Moreover, there were no new safety concerns [46–48]. Contrarily, FAc implant seems to be better tolerated in real-world studies than in randomised control trials.

In the next section we will aim to address some of the main concerns arisen from the clinical management on DMO eyes treated with the 0.2 µg/day FAc implant over the last decade.

### DMO patients who do not adequately respond to treatment

Insufficiently responsive patients can be defined according to their visual response or their anatomical response [49, 50].

Based on their visual response:

- A visual acuity [VA] improvement ≤5 letters ETDRS.

Based on their anatomic response:

- Central retinal thickness [CRT] reduction [assessed by optical coherence tomography, OCT] <20%.

Regarding the type of treatment, approximately one third [range 28% to 40%] of patients with DMO may be considered non-responders to anti-VEGF [49, 51–55], whilst the reported rate of anatomic non-responders to dexamethasone intravitreal implant [DEX-i] varied between 14% at month 6 [56] in the PREDIAMEX study and 12.0% in the GRADE-DME Study at month 4 [57].

Although VEGF has been identified as a key contributor factor in the development of DMO [58, 59], it does not play a fundamental role in all DMO cases. In fact, in eyes who did not adequately respond to anti-VEGF treatment, there were higher levels of inflammatory biomarkers, such as IL-6, IL-8, tumour necrosis factor receptor [TNFR]-1 and -2, and matrix metalloproteinase [MMP]-9 [60].

### Clinical trials versus real-life outcomes: Are the clinical practice outcomes comparable to those obtained in clinical trials?

Despite numerous RCTs have proven the efficacy and safety of anti-VEGF drugs [61], results obtained in real life have not been matching with those reported in RCTs [62, 63].

Although RCTs represent the highest level of evidence [64], application of their outcomes to clinical practice often is not straightforward [65]. The strict inclusion and exclusion criteria used to select patients for inclusion in RCTs is not compatible with day-to-day practice where treatment is offered to a much wider range of patients [65].

According to Ciulla et al. [62], in eyes with DMO who underwent treatment with anti-VEGF, visual outcomes in the real clinical setting were lower to those reported in RCTs by ~1 line of VA at 1 year. Different factors might be associated with this finding, including receiving fewer anti-VEGF injections [i.e., undertreatment]. In addition, DMO patient eyes may have well-preserved baseline VA, which limits the VA improvement due to a ceiling effect; or, on the contrary, DMO eyes included in real-life studies might have an extremely poor baseline VA [i.e., an advanced DMO], which also may limit the VA improvement and the final VA [62]. The disconnect between RCTs and real-life outcomes is a major issue with anti-VEGF treatments, mainly due to non-compliance with the tight schedule of monitoring and injections that is mandatory to achieve best outcomes [62, 63].

Moreover, the differences between the RCTs and real-world studies outcomes can be greater in the low- to lower-middle-income countries, since they are faced with additional barriers [63].

When comparing the visual outcomes of anti-VEGF with those of DEX-i, it has been suggested that in real-world studies DEX-i provided superior VA improvements than anti-VEGF [66].

Regarding FAc implant, real-world studies have achieved similar visual and anatomic outcomes than RCTs but better safety outcomes [34].

### Can we anticipate a clinical response to anti-VEGF therapy?

It seems that the rate of non-responders to anti-VEGF does not really vary over time, which clearly suggests that is not dependent on the number of injections [51].

This finding may be explained by the fact that despite the vitreous levels of VEGF was significantly greater in patients with DMO than in control subjects, ~20–30% of patients with DMO had normal vitreous levels of VEGF [67].

Moreover, concentration of VEGF in aqueous humour might predict the response to anti-VEGF [60]. Patients who responded adequately and rapidly to anti-VEGF had higher aqueous humour concentration of VEGF than non-responders. While patients who responded to corticosteroids but not to anti-VEGF therapy showed significantly lower levels of VEGF than patients with rapid response [60].

Finally, patients with an excellent response to anti-VEGF had increased baseline VEGF and decreased monocyte chemotactic protein-1 concentrations in aqueous humour compared with non-responders, while aqueous humour concentrations of Interleukin-6 were greater among non-responders to anti-VEGF [68].

However, according to data from protocol T, it seemed that maintaining anti-VEGF treatment for 24 weeks might have positive outcomes on DMO resolution [49].

Another factor to consider is the duration of macular oedema, since VA outcomes are worse in DMO with a longer duration [69] and persistence of DMO was a negative prognostic factor for long-term visual acuity improvement [70]. In addition, delaying DMO treatment has been associated with worse visual outcomes [71].

Regarding whether it is possible to predict a functional and/or an anatomic response to anti-VEGF therapy, it has been recently published that greater baseline BCVA and longer DR duration were negative predictors of visual outcomes, whereas aflibercept treatment and an early functional response were positive predictors for vision improvement [72]. A pre-treatment thicker macula and an early CRT reduction were predictors for better anatomic outcomes, while baseline HbA1c value was a negative predictor for CRT reduction [72].

When deciding to switch the current treatment, one question that the clinicians often face is: what would be the treatment of choice?

#### Does it make sense to switch from one anti-VEGF to other one?

It has been previously reported that aflibercept provided better functional and anatomic outcomes than bevacizumab and ranibizumab [73]. However, such difference was not considered clinically relevant since outcomes critically depended on baseline parameters [73]. Moreover, if we consider only real-world data, aflibercept has not provided better functional results than bevacizumab or than ranibizumab [74].

Although there is evidence suggesting that switching anti-VEGF drug to another anti-VEGF agent may provide favourable anatomical and functional outcomes, the lack of a control group that maintains the initial treatment greatly limits the findings obtained [75]. Moreover, the majority of non-responders are explained by a quite normal VEGF ocular concentration. Thus, switching to another anti-VEGF would not improve outcomes. Pooled data from the YOSEMITE and RHINE trials revealed comparable efficacy and safety outcomes among patients receiving either faricimab 6.0 mg every 8 weeks, faricimab 6.0 mg personalised to their treatment intervals, or aflibercept 2.0 mg every 8 weeks, both at the one-year and two-years of treatment [76, 77]. Additionally, findings from real-world data, suggested that faricimab might be an effective therapy for both treatment-naïve and previously treated DMO patients [78].

Regarding brolucizumab, both KESTREL [patients were randomly assigned to receive either brolucizumab 3 mg, brolucizumab 6 mg or aflibercept 2 mg] and KITE [patients were randomly assigned to receive either brolucizumab 6 mg or aflibercept 2 mg] trials found that, at week 100, clinical outcomes achieved with brolucizumab 6 mg were comparable to those of aflibercept in both studies [79].

However, the current evidence is limited, lacking data from long-term real-life studies.

#### Does it make sense to switch from one anti-VEGF to a corticosteroid intravitreal implant?

There is evidence supporting the effectiveness and safety of switching to DEX-i in DMO eyes with an insufficient response to anti-VEGF [55, 80–87].

With regards, FAc implant, since it has been approved for treating eyes who do not adequately respond to other therapies [18, 19], both the results of the RCTs and those of the real-world studies, mentioned throughout this article, have demonstrated that switching to the 0.19 mg FAc implant provided better visual and anatomic outcomes than the previous treatments [20–45]. Indeed, according to the results of the European IRISS registry study, early switch was associated not only with better visual outcomes, but also with lower incidence of IOP-related events [39] (See Fig. S2).

### Is the possibility of ocular hypertension onset a reason not to use 0.19 mg FAc implant?

Tables S2 and S3 summarise the incidence of IOP-related AEs in RCTs and real-world studies, respectively.

Although the proportion of IOP-related AEs is relatively important, in most cases they are perfectly controlled with topical ocular hypotensive medications, and the proportion of eyes requiring any type of glaucoma surgery is much lower in the real-world studies, than in the RCTs [see Tables S2 and S3].

Moreover, elevation of IOP has been associated with both DEX-i [88–92] and anti-VEGF [93, 94] treatments. In fact, a patient with no pressure warning on 2 to 3 DEX-i has an 85–90% chance of maintaining the same tolerance profile on FAc implant [26, 30, 31]. Whereas a patient who has had an increase in IOP [IOP > 25 mmHg] on DEX-i is 20 times more likely to maintain the same tolerance profile on FAc implant [46, 95].

According to the recommendations of a European consensus, FAc implant can be administered in eyes with a previous history of OHT after treatment with DEX-i if the OHT is controlled with a dual therapy at maximum [although monotherapy is preferable] and the patient experienced an IOP elevation ≤10 mmHg [96].

The 2023 French guidelines on ocular hypertension (OHT) and intravitreal steroid injections emphasise optimised management strategies for corticosteroid-induced OHT, reported in approximately one-third of cases [97]. These guidelines stress the importance of thorough IOP evaluation prior to corticosteroid implant administration and tailored, implant-specific IOP monitoring during follow-up. The recommendations also highlight using dexamethasone implants as a preliminary test to evaluate pressure tolerance before transitioning to fluocinolone implants. For refractory cases, selective laser trabeculoplasty is proposed as a complementary approach to conventional hypotensive therapies. This framework aims to enhance treatment safety and efficacy [97].

### What is the place of the 0.19 mg FAc implant in the DMO therapeutic algorithm?

FAc implant and DEX-i have different pharmacokinetics and this entails two different objectives. While DEX-i has a burst effect releases immediately a significant dose of dexamethasone once injected [with a peak of 1110 ng/g eight weeks later] [98, 99]; the FAc implant delivers well controlled but lower concentrations of fluocinolone acetonide with an initial release of 1.26 ng/g the first few days followed by a sustained release of 0.261 ng/g at 3 months and for 3 years [13, 99].

In real-world studies, ~60% of patients treated with the FAc implant for DMO required additional therapeutic interventions within two years [25]. However, this should not be interpreted as a failure of the FAc implant but rather as its role as a background treatment strategy [34, 83]. Initiating therapy with a short-acting intravitreal steroid (such as dexamethasone implant) might be recommended to achieve rapid therapeutic effects, followed by FAc implantation to maintain long-term benefits [18, 99]. This sequential approach also facilitates a more accurate prediction of OHT risk compared to topical dexamethasone, given the pharmacokinetic differences between intravitreal and topical administration [83].

We can make an analogy with the treatment of migraine, in which attack drugs are used with the objective to quickly control the pain; while maintenance treatment is used to decrease the frequency and intensity of attacks, reduce the need for attack treatments, and improve the patient’s quality of life] [100].

Following a similar model, the treatment of DMO would include two fundamental strategies. The first is to resolve, as soon as possible, the macular oedema, i.e., an “attack treatment”; while the second strategy aims to reduce the frequency and intensity of relapses, i.e., “a maintenance/background treatment” [74, 101].

Based on the current evidence, therefore, the role of 0.19 mg FAc implant may be changed from “treating chronic and recalcitrant DMO” to “reduce the therapeutic burden”; “preventing DMO recurrences”; and “reducing anatomic fluctuations” (See Fig. S3).

One drawback of short-term treatments like, for example, anti-VEGF injections is their association with increased retinal thickness variability (RTV). These fluctuations lead to inconsistencies in disease control levels between injections and may contribute to declining visual acuity outcomes over time, despite their effectiveness [102, 103].

A post hoc analysis of the PALADIN study revealed that the 0.19 mg FAc implant reduced significantly the RTV over 36 months. Furthermore, this RTV reduction was associated to a greater proportion of patients achieving a dry retina. Moreover, RTV was directly correlated with improved visual outcomes (the lower RTV the greater BCVA improvement) and reduced treatment requirements [103].

Therefore, in the treatment of DMO, 0.19 mg FAc implant would be considered as a maintenance/background treatment, which aims to prevent recurrences of DMO and subsequent vision loss.

According to everything seen so far, the ideal candidate DMO patient to receive treatment with the 0.19 FAc implant would be one who:

- Has shown a good safety profile [regarding to IOP] with DEX-I [33, 36].
- Has achieved a good functional and anatomical response under DEX-I [46, 95].
- Has recently been diagnosed with chronic DMO [39, 104].
- An early switch is indicated [35, 36].
- Would be desirable to reduce anatomical fluctuations [36, 105].
- Would be desirable [either on the part of physician and/or patient] to reduce the therapeutic burden [25, 31, 33, 35, 36, 39, 40, 46, 95, 104–107].

According to the results of a retrospective cohort study that evaluated the effectiveness and safety of 0.19 mg FAc implant over 5-years, 68% of eyes had stabilised or improved VA at 5 years from inclusion [106]. In addition, retinal thickness was reduced by 32% at year-1 and remained stable after 5 years of follow-up. Moreover, at 5 years post-FAc implant, 42% of patients did not require additional treatment [106].

The most important aspects in the clinical management of patients with DMO learned during the 10 years of availability of the 0.19 mg FAc implant have been summarised in the Table 2.

**Table 2:** Ten lessons in the clinical management of diabetic macular oedema (DMO): pearls, pitfalls, and myths.

Question	Answer
What is a non-responder in DMO?	VA gain <5 letters & OCT thickness reduction <20%
What is the percentage of non-responders with anti-VEGF and steroids? • How do we explain non-responders in DMO?	1/3 for anti-VEGF & less than 1/5 with FAc implant and DEX-i • Partly explained because VEGF level is almost normal in one third of DMO
What visual gain can we expect in real life? • Can we reproduce interventional outcomes in observational real-life studies with anti-VEGF with DEX-i and 0.19 mg FAc implant?	Real-life outcomes with 0.19 mg FAc implant and DEX-i (≈ + 9 letters) appear to be better to real-life outcomes with anti-VEGF ( ≈ + 5 letters), regardless of baseline VA level, partly explained by much less anti-VEGF injections in daily practice • No, we cannot with anti-VEGF contrarily to DEX-i and 0.19 mg FAc implant
When should a patient be switched? • Does the duration of oedema matter? • If you switch, which family of agents should you choose next?	The rate of non-responder with anti-VEGF (1/3) does not really vary over time, meaning that is not dependent of the number of injections. • Yes, it does. VA outcomes are worse in DMO with longer duration • Change to another family like steroids
At what point can we predict a functional response to anti-VEGF therapy?	Switch early after the first 3 to 6 injections (within the first 5 to 6 months after treatment initiation), if required
Is ocular hypertension common? • Ocular hypertension exists also with DEX-I … and anti-VEGF	1/3 0.19 mg FAc implant-induced OHT during follow-up and better tolerance in real life with fewer hypotonizing surgeries • ≈ 20% IOP > 25 mmHg in DMO with DEX-I; 5–10% with anti-VEGF at 1 year
Is the DEX test predictive of IOP related events following treatment with 0.19 mg FAc implant? • Can we inject with 0.19 mg FAc implant a patient who presented with ocular hypertension with dexamethasone?	YES, a patient with no pressure warning on 2 to 3 DEX-I has an 85–90% chance of maintaining the same tolerance profile on 0.19 mg FAc implant. • YES, if ocular hypertension was controlled with a dual therapy at maximum and the patient was a poor or intermediate responder
How do you introduce 0.19 mg FAc implant to a patient? • How do you inform a patient on how 0.19 mg FAc implant works? expectation of response, additional treatments and that they need to come back and check their vision and IOP?	Efficacy is obtained in few months; No risk of disaster with OHT if quarterly follow-up is respected; 1/3 of patients require additional treatment but there is always a significant spacing of injections observed, permitting to reduce the therapeutic burden
Which DMO algorithm do you use with 19 mg FAc implant?	Two different implants with 2 different objectives: • Background treatment: 19 mg FAc implant to prevent DMO recurrence • Attack treatment: DEX-i to treat DMO
What has been your greatest learnings from using 19 mg FAc implant for the treatment of DMO?	Useful to decrease the anatomical fluctuations and the total number of injections

*DMO* Diabetic macular oedema, *VA* Visual acuity, *OCT* Optical coherence tomography, *anti-VEGF* Vascular endothelial growth factor inhibitors, *FAc* Fluocinolone acetonide intravitreal, *DEX-i* Dexamethasone intravitreal implant, *OHT* Ocular hypertension.

## Conclusions

Since inflammation is a key factor contributing to the development of DMO, targeting inflammation with intravitreal corticosteroid implants has proven to be effective due mainly to a more comprehensive mechanism of action compared to the targeted therapy with anti-VEGF.

If we consider the treatment of DMO, 0.19 mg FAc implant would be indicated as a maintenance/background treatment to prevent recurrences of DMO and subsequent vision loss, with a prolonged durability of 2 to 3 years.

The main safety concerns of the 0.19 mg FAc implant are cataract and elevation of IOP. Although the incidence of IOP related AEs with 0.19 mg FAc is relatively high [although lower in real-world studies than in RCTs], in most cases it is controlled wit topical hypotensive medications.

Finally, 0.19 mg FAc implant sustained release reduces significantly the treatment burden.

## Summary

### What is known about this topic

- Diabetic macular oedema (DMO) is a significant cause of vision impairment among diabetic patients, requiring long-term treatment to prevent disease progression.
- Corticosteroid implants, such as the 0.19 mg fluocinolone acetonide (FAc) implant, have been introduced as long-lasting therapeutic options for DMO management.
- While anti-VEGF injections remain the first-line treatment, corticosteroid implants are beneficial for patients with chronic or refractory DMO who do not respond adequately to other therapies.
- The main adverse effects associated with corticosteroid implants include intraocular pressure (IOP) elevation and cataract formation, which may require additional interventions.

### What this study adds

- The 0.19 mg FAc implant demonstrates sustained efficacy in improving visual acuity and reducing central retinal thickness over an extended follow-up period, with benefits lasting up to 3 years.
- Real-world evidence suggests that the FAc implant is well-tolerated, with a lower incidence of IOP-related complications compared to randomized controlled trials.
- A proportion of patients treated with the FAc implant do not require additional intravitreal injections, reducing the overall treatment burden.
- The findings support the role of FAc implants as maintenance therapy in DMO, preventing disease recurrence and stabilizing retinal anatomy.

## Supplementary information

Supplementary Material

## Data Availability

Data sharing not applicable to this article as no datasets were generated or analysed during the current study.

## References

[1] World Health Organization. Diabetes. [April 5, 2023]. Available in: https://www.who.int/news-room/fact-sheets/detail/diabetes Last accessed April, 2024.

[2] Li JQ, Welchowski T, Schmid M, Letow J, Wolpers AC, et al. Retinal Diseases inEurope: Prevalence, incidence and healthcare needs. Available in: https://miloftalmica.it/wp-content/uploads/2021/07/Euretina-Retinal-Diseases.pdf Last accessedApril, 2024.

[3] Sun H, Saeedi P, Karuranga S, Pinkepank M, Ogurtsova K, Duncan BB, et al. IDF Diabetes Atlas: Global, regional and country-level diabetes prevalence estimates for 2021 and projections for 2045. Diabetes Res Clin Pract. 2022;183:109119. 34879977 10.1016/j.diabres.2021.109119 PMC11057359

[4] Williams R, Karuranga S, Malanda B, Saeedi P, Basit A, Besançon S, et al. Global and regional estimates and projections of diabetes-related health expenditure: results from the International Diabetes Federation Diabetes Atlas, 9th edition. Diabetes Res Clin Pract. 2020;162:108072. 32061820 10.1016/j.diabres.2020.108072

[5] Teo ZL, Tham YC, Yu M, Chee ML, Rim TH, Cheung N, et al. Global prevalence of diabetic retinopathy and projection of burden through 2045: systematic review and meta-analysis. Ophthalmology. 2021;128:1580–91. 33940045 10.1016/j.ophtha.2021.04.027

[6] Zhang X, Zeng H, Bao S, Wang N, Gillies MC. Diabetic macular edema: new concepts in patho-physiology and treatment. Cell Biosci. 2014;4:27. 24955234 10.1186/2045-3701-4-27 PMC4046142

[7] Das A, McGuire PG, Rangasamy S. Diabetic macular edema: pathophysiology and novel therapeutic targets. Ophthalmology. 2015;122:1375–94. 25935789 10.1016/j.ophtha.2015.03.024

[8] Romero-Aroca P, Baget-Bernaldiz M, Pareja-Rios A, Lopez-Galvez M, Navarro-Gil R, Verges R. Diabetic macular edema pathophysiology: vasogenic versus inflammatory. J Diabetes Res. 2016;2016:2156273. 27761468 10.1155/2016/2156273 PMC5059543

[9] Roy S, Kern TS, Song B, Stuebe C. Mechanistic insights into pathological changes in the diabetic retina: implications for targeting diabetic retinopathy. Am J Pathol. 2017;187:9–19. 27846381 10.1016/j.ajpath.2016.08.022 PMC5225303

[10] Daruich A, Matet A, Moulin A, Kowalczuk L, Nicolas M, Sellam A, et al. Mechanisms of macular edema: beyond the surface. Prog Retin Eye Res. 2018;63:20–68. 29126927 10.1016/j.preteyeres.2017.10.006

[11] Bunch KL, Abdelrahman AA, Caldwell RB, Caldwell RW. Novel Therapeutics for diabetic retinopathy and diabetic macular edema: a pathophysiologic perspective. Front Physiol. 2022;13:831616. 35250632 10.3389/fphys.2022.831616 PMC8894892

[12] Costa C, Incio J, Soares R. Angiogenesis and chronic inflammation: cause or consequence? Angiogenesis 2007;10:149–66. 17457680 10.1007/s10456-007-9074-0

[13] Sheemar A, Soni D, Takkar B, Basu S, Venkatesh P. Inflammatory mediators in diabetic retinopathy: deriving clinicopathological correlations for potential targeted therapy. Indian J Ophthalmol. 2021;69:3035–49. 34708739 10.4103/ijo.IJO_1326_21 PMC8725076

[14] Schmidt-Erfurth U, Garcia-Arumi J, Bandello F, Berg K, Chakravarthy U, Gerendas BS, et al. Guidelines for the management of diabetic macular edema by the European Society of Retina Specialists (EURETINA). Ophthalmologica. 2017;237:185–222. 28423385 10.1159/000458539

[15] Barham R, El Rami H, Sun JK, Silva PS. Evidence-based treatment of diabetic macular edema. Semin Ophthalmol. 2017;32:56–66. 28060586 10.1080/08820538.2016.1228388

[16] Kim EJ, Lin WV, Rodriguez SM, Chen A, Loya A, Weng CY. Treatment of diabetic macular edema. Curr Diab Rep. 2019;19:68. 31359157 10.1007/s11892-019-1188-4

[17] Singh RP, Elman MJ, Singh SK, Fung AE, Stoilov I. Advances in the treatment of diabetic retinopathy. J Diabetes Complications 2019;33:107417. 31669065 10.1016/j.jdiacomp.2019.107417

[18] Campochiaro PA, Nguyen QD, Hafiz G, Bloom S, Brown DM, Busquets M, et al. Aqueous levels of fluocinolone acetonide after administration of fluocinolone acetonide inserts or fluocinolone acetonide implants. Ophthalmology. 2013;120:583–7. 23218184 10.1016/j.ophtha.2012.09.014

[19] Summary of product characteristics ILUVIEN 190 micrograms intravitreal implant in applicator. Available at: https://www.medicines.org.uk/emc/product/3061/smpc#gref Last accessed April 12, 2024.

[20] Campochiaro PA, Brown DM, Pearson A, Ciulla T, Boyer D, Holz FG, et al. Long-term benefit of sustained-delivery fluocinolone acetonide vitreous inserts for diabetic macular edema. Ophthalmology. 2011;118:626–635.e2. 21459216 10.1016/j.ophtha.2010.12.028

[21] Campochiaro PA, Brown DM, Pearson A, Chen S, Boyer D, Ruiz-Moreno J, et al. Sustained delivery fluocinolone acetonide vitreous inserts provide benefit for at least 3 years in patients with diabetic macular edema. Ophthalmology. 2012;119:2125–32. 22727177 10.1016/j.ophtha.2012.04.030

[22] Cunha-Vaz J, Ashton P, Iezzi R, Campochiaro P, Dugel PU, Holz FG, et al. Sustained delivery fluocinolone acetonide vitreous implants: long-term benefit in patients with chronic diabetic macular edema. Ophthalmology. 2014;121:1892–903. 24935282 10.1016/j.ophtha.2014.04.019

[23] Alfaqawi F, Lip PL, Elsherbiny S, Chavan R, Mitra A, Mushtaq B. Report of 12-months efficacy and safety of intravitreal fluocinolone acetonide implant, for the treatment of chronic diabetic macular oedema: a real-world result in the United Kingdom. Eye. 2017;31:650–6. 28106887 10.1038/eye.2016.301 PMC5396008

[24] Bailey C, Chakravarthy U, Lotery A, Menon G, Talks J, Medisoft Audit Group. Real-world experience with 0.2 μg/day fluocinolone acetonide intravitreal implant (ILUVIEN) in the United Kingdom. Eye. 2017;31:1707–15. 28737758 10.1038/eye.2017.125 PMC5733285

[25] Fusi-Rubiano W, Mukherjee C, Lane M, Tsaloumas MD, Glover N, Kidess A, et al. Treating Diabetic Macular Oedema (DMO): real world UK clinical outcomes for the 0.19 mg Fluocinolone Acetonide intravitreal implant (Iluvien™) at 2 years. BMC Ophthalmol. 2018;18:62. 29486754 10.1186/s12886-018-0726-1 PMC6389097

[26] Eaton A, Koh SS, Jimenez J, Riemann CD. The USER Study: a chart review of patients receiving a 0.2 µg/day fluocinolone acetonide implant for diabetic macular edema. Ophthalmol Ther. 2019;8:51–62. 30560505 10.1007/s40123-018-0155-5 PMC6393252

[27] Young JF, Walkden A, Stone A, Mahmood S. Clinical effectiveness of intravitreal Fluocinolone Acetonide (FAc) (ILUVIENTM) in patients with Diabetic Macular Oedema (DMO) refractory to prior therapy: the manchester experience. Ophthalmol Ther. 2019;8:477–84. 31309417 10.1007/s40123-019-0197-3 PMC6692423

[28] Augustin AJ, Bopp S, Fechner M, Holz F, Sandner D, Winkgen AM, et al. Three-year results from the Retro-IDEAL study: real-world data from diabetic macular edema (DME) patients treated with ILUVIEN® (0.19mg fluocinolone acetonide implant). Eur J Ophthalmol. 2020;30:382–91. 30884972 10.1177/1120672119834474 PMC7079293

[29] Chakravarthy U, Taylor SR, Koch FHJ, Castro de Sousa JP, Bailey C. ILUVIEN Registry Safety Study (IRISS) Investigators Group. Changes in intraocular pressure after intravitreal fluocinolone acetonide (ILUVIEN): real-world experience in three European countries. Br J Ophthalmol. 2019;103:1072–7. 30242062 10.1136/bjophthalmol-2018-312284 PMC6678053

[30] Mansour SE, Kiernan DF, Roth DB, Eichenbaum D, Holekamp NM, Kaba S, et al. Two-year interim safety results of the 0.2 µg/day fluocinolone acetonide intravitreal implant for the treatment of diabetic macular oedema: the observational PALADIN study. Br J Ophthalmol. 2021;105:414–9. 32461262 10.1136/bjophthalmol-2020-315984 PMC7907551

[31] Panos GD, Arruti N, Patra S. The long-term efficacy and safety of fluocinolone acetonide intravitreal implant 190 μg (ILUVIEN®) in diabetic macular oedema in a multi-ethnic inner-city population. Eur J Ophthalmol. 2021;31:620–9. 31906704 10.1177/1120672119898414

[32] Mushtaq B, Bhatnagar A, Palmer H. Real-world outcomes in diabetic macular edema for the 0.2 µg/Day fluocinolone acetonide implant: case series from the Midlands, UK. Clin Ophthalmol. 2021;15:2935–43. 34262254 10.2147/OPTH.S283561 PMC8274235

[33] Bailey C, Chakravarthy U, Lotery A, Menon G, Talks J, Medisoft Audit Group. Extended real-world experience with the ILUVIEN® (fluocinolone acetonide) implant in the United Kingdom: 3-year results from the Medisoft® audit study. Eye. 2022;36:1012–8. 33972705 10.1038/s41433-021-01542-w PMC8107780

[34] Kodjikian L, Baillif S, Creuzot-Garcher C, Delyfer MN, Matonti F, Weber M, et al. Real-world efficacy and safety of fluocinolone acetonide implant for diabetic macular edema: a systematic review. Pharmaceutics. 2021;13:72. 33430389 10.3390/pharmaceutics13010072 PMC7827527

[35] Mathis T, Papegaey M, Ricard C, Rezkallah A, Matonti F, Sudhalkar A, et al. Efficacy and safety of intravitreal fluocinolone acetonide implant for chronic diabetic macular edema previously treated in real- life practice: the REALFAc study. Pharmaceutics. 2022;14:723. 35456557 10.3390/pharmaceutics14040723 PMC9025285

[36] Singer MA, Sheth V, Mansour SE, Coughlin B, Gonzalez VH. Three-Year Safety and efficacy of the 0.19-mg fluocinolone acetonide intravitreal implant for diabetic macular edema: the PALADIN Study. Ophthalmology. 2022;129:605–13. 35063472 10.1016/j.ophtha.2022.01.015

[37] Elbarky AM. Effectiveness and tolerability of the fluocinolone acetonide implant in patients with diabetic macular edema in UAE: 12-Month results. Eur J Ophthalmol. 2021;31:3196–202. 33426902 10.1177/1120672120982948

[38] Fallico M, Maugeri A, Lotery A, Longo A, Bonfiglio V, Russo A, et al. Fluocinolone acetonide vitreous insert for chronic diabetic macular oedema: a systematic review with meta-analysis of real-world experience. Sci Rep. 2021;11:4800. 33637841 10.1038/s41598-021-84362-y PMC7910468

[39] Khoramnia R, Peto T, Koch F, Taylor SR, Castro de Sousa JP, Hill L, et al. ILUVIEN Registry Safety Study (IRISS) Investigators Group. Safety and effectiveness of the fluocinolone acetonide intravitreal implant (ILUVIEN): 3-year results from the European IRISS registry study. Br J Ophthalmol. 2023;107:1502–8. 35840291 10.1136/bjo-2022-321415 PMC10579189

[40] Merrill PT, Holekamp N, Roth D, Kasper J, Grigorian R. PALADIN Study Group. The 0.19-mg fluocinolone acetonide intravitreal implant reduces treatment burden in diabetic macular edema. Am J Ophthalmol. 2023;248:16–23. 36223849 10.1016/j.ajo.2022.09.017

[41] Roth DB, Eichenbaum D, Malik D, Radcliffe NM, Cutino A, Small KW, et al. The 0.19-mg fluocinolone acetonide intravitreal implant for diabetic macular edema: intraocular pressure-related effects over 36 Months. Ophthalmol Retina. 2024;8:49–54. 37586482 10.1016/j.oret.2023.08.004

[42] Ruiz-Moreno JM, Adán A, Lafuente M, Asencio Durán M, Arias Barquet L, García Layana A, et al. Effectiveness and safety of fluocinolone acetonide intravitreal implant in diabetic macular edema patients considered insufficiently responsive to available therapies (REACT): a prospective, non-randomized, and multicenter study. Int Ophthalmol. 2023;43:4639–49. 37697082 10.1007/s10792-023-02864-2 PMC10724319

[43] Rousseau N, Lebreton O, Masse H, Maucourant Y, Pipelart V, Clement M, et al. Fluocinolone acetonide implant injected 1 month after dexamethasone implant for diabetic macular oedema: the ILUVI1MOIS Study. Ophthalmol Ther. 2023;12:2781–92. 37369907 10.1007/s40123-023-00749-2 PMC10441852

[44] Yang Y, Bailey C, Holz FG, Eter N, Weber M, Baker C, et al. Long-term outcomes of phakic patients with diabetic macular oedema treated with intravitreal fluocinolone acetonide (FAc) implants. Eye. 2015;29:1173–80. 26113503 10.1038/eye.2015.98 PMC4565956

[45] Parrish RK 2nd, Campochiaro PA, Pearson PA, Green K, Traverso CE; FAME Study Group. Characterization of Intraocular pressure increases and management strategies following treatment with fluocinolone acetonide intravitreal implants in the FAME trials. Ophthal Surg Lasers Imaging Retina. 2016;47:426–35. 10.3928/23258160-20160419-05 27183546

[46] Capone L, Airaghi P, Aragona P, Castellino N, Cicinelli MV, Ciucci F, et al. Real-world experience with fluocinolone acetonide intravitreal implant in patients with diabetic macular edema. Eur J Ophthalmol. 2024:11206721241235266. 10.1177/11206721241235266. 10.1177/11206721241235266 38396370

[47] Lebrize S, Arnould L, Bourredjem A, Busch C, Rehak M, Massin P, et al. Intraocular pressure changes after intravitreal fluocinolone acetonide implant: results from four European Countries. Ophthalmol Ther. 2022;11:1217–29. 35426623 10.1007/s40123-022-00504-z PMC9114211

[48] Teixeira C, Pessoa B, Ruão M, Sousa JPC, Penas S, Silva R, et al. ILUVIEN® in diabetic macular edema that persists or recurs despite treatment: Results from the Retina.pt® RIVER audit. Eur J Ophthalmol. 2023:11206721231217525. 10.1177/11206721231217525.

[49] Bressler NM, Beaulieu WT, Glassman AR, Blinder KJ, Bressler SB, Jampol LM, et al. Diabetic retinopathy clinical research network. persistent macular thickening following intravitreous Aflibercept, Bevacizumab, or Ranibizumab for Central-involved diabetic macular edema with vision impairment: a secondary analysis of a randomized clinical trial. JAMA Ophthalmol. 2018;136:257–69. 29392288 10.1001/jamaophthalmol.2017.6565 PMC5885906

[50] Udaondo P, Adan A, Arias-Barquet L, Ascaso FJ, Cabrera-López F, Castro-Navarro V, et al. Challenges in diabetic macular edema management: an expert consensus report. Clin Ophthalmol. 2021;15:3183–95. 34349495 10.2147/OPTH.S320948 PMC8327476

[51] Diabetic Retinopathy Clinical Research Network, Elman MJ, Aiello LP, Beck RW, Bressler NM, Bressler SB, Edwards AR, et al. Randomized trial evaluating ranibizumab plus prompt or deferred laser or triamcinolone plus prompt laser for diabetic macular edema. Ophthalmology. 2010;117:1064–1077.e35. 20427088 10.1016/j.ophtha.2010.02.031 PMC2937272

[52] Mitchell P, Bandello F, Schmidt-Erfurth U, Lang GE, Massin P, Schlingemann RO, et al. The RESTORE study: ranibizumab monotherapy or combined with laser versus laser monotherapy for diabetic macular edema. Ophthalmology. 2011;118:615–25. 21459215 10.1016/j.ophtha.2011.01.031

[53] Bressler SB, Ayala AR, Bressler NM, Melia M, Qin H, Ferris FL 3rd, et al. Diabetic retinopathy clinical research network. persistent macular thickening after ranibizumab treatment for diabetic macular edema with vision impairment. JAMA Ophthalmol. 2016;134:278–85. 26746868 10.1001/jamaophthalmol.2015.5346 PMC4786449

[54] Gonzalez VH, Campbell J, Holekamp NM, Kiss S, Loewenstein A, Augustin AJ, et al. Early and long-term responses to anti-vascular endothelial growth factor therapy in diabetic macular edema: analysis of protocol I Data. Am J Ophthalmol. 2016;172:72–79. 27644589 10.1016/j.ajo.2016.09.012

[55] Massin P, Creuzot-Garcher C, Kodjikian L, Girmens JF, Delcourt C, Fajnkuchen F, et al. Real-world outcomes with Ranibizumab 0.5 mg in patients with visual impairment due to diabetic macular Edema: 12-Month results from the 36-Month BOREAL-DME Study. Ophthalmic Res. 2019;62:101–10. 30928985 10.1159/000497406

[56] Bellocq D, Akesbi J, Matonti F, Vartin C, Despreaux R, Comet A, et al. The Pattern of recurrence in diabetic macular edema treated by dexamethasone implant: the PREDIAMEX Study. Ophthalmol Retina. 2018;2:567–73. 31047610 10.1016/j.oret.2017.10.016

[57] Rodríguez-Valdés PJ, Rehak M, Zur D, Sala-Puigdollers A, Fraser-Bell S, Lupidi M, et al. GRAding of functional and anatomical response to DExamethasone implant in patients with Diabetic Macular Edema: GRADE-DME Study. Sci Rep. 2021;11:4738. 33637772 10.1038/s41598-020-79288-w PMC7910444

[58] Witmer AN, Vrensen GF, Van Noorden CJ, Schlingemann RO. Vascular endothelial growth factors and angiogenesis in eye disease. Prog Retin Eye Res. 2003;22:1–29. 12597922 10.1016/s1350-9462(02)00043-5

[59] Wautier JL, Wautier MP. Vascular permeability in diseases. Int J Mol Sci. 2022;23:3645. 35409010 10.3390/ijms23073645 PMC8998843

[60] Udaondo P, Hernández C, Briansó-Llort L, García-Delpech S, Simó-Servat O, Simó R. Usefulness of Liquid Biopsy Biomarkers from Aqueous Humor in Predicting Anti-VEGF Response in Diabetic Macular Edema: Results of a Pilot Study. J Clin Med. 2019;8:1841. 31684007 10.3390/jcm8111841 PMC6912573

[61] Virgili G, Parravano M, Evans JR, Gordon I, Lucenteforte E. Anti-vascular endothelial growth factor for diabetic macular oedema: a network meta-analysis. Cochrane Database Syst Rev. 2018;10:CD007419. 30325017 10.1002/14651858.CD007419.pub6 PMC6517135

[62] Ciulla TA, Bracha P, Pollack J, Williams DF. Real-world outcomes of anti-vascular endothelial growth factor therapy in diabetic macular Edema in the United States. Ophthalmol Retina 2018;2:1179–87. 31047187 10.1016/j.oret.2018.06.004

[63] Sam-Oyerinde OA, Patel PJ. Real-world outcomes of Anti-VEGF therapy in diabetic macular oedema: barriers to treatment success and implications for low/lower-middle-income Countries. Ophthalmol Ther. 2023;12:809–26. 36821027 10.1007/s40123-023-00672-6 PMC10011234

[64] Burns PB, Rohrich RJ, Chung KC. The levels of evidence and their role in evidence-based medicine. Plast Reconstr Surg. 2011;128:305–10. 21701348 10.1097/PRS.0b013e318219c171 PMC3124652

[65] Zarbin M. Real Life Outcomes vs. Clinical Trial Results. J Ophthalmic Vis Res. 2019;14:88–92. 30820292 10.4103/jovr.jovr_279_18 PMC6388532

[66] Kodjikian L, Bellocq D, Mathis T. Pharmacological management of diabetic macular edema in real-life observational studies. Biomed Res Int. 2018;2018:8289253. 30246026 10.1155/2018/8289253 PMC6136521

[67] Funatsu H, Yamashita H, Ikeda T, Mimura T, Eguchi S, Hori S. Vitreous levels of interleukin-6 and vascular endothelial growth factor are related to diabetic macular edema. Ophthalmology. 2003;110:1690–6. 13129863 10.1016/S0161-6420(03)00568-2

[68] Abraham JR, Wykoff CC, Arepalli S, Lunasco L, Yu HJ, Hu M, et al. Aqueous Cytokine expression and higher order OCT biomarkers: assessment of the anatomic-biologic bridge in the IMAGINE DME study. Am J Ophthalmol. 2021;222:328–39. 32896498 10.1016/j.ajo.2020.08.047 PMC9719825

[69] Gardner TW, Larsen M, Girach A, Zhi X. Protein Kinase C diabetic retinopathy Study (PKC-DRS2) Study Group. Diabetic macular oedema and visual loss: relationship to location, severity and duration. Acta Ophthalmol. 2009;87:709–13. 19817721 10.1111/j.1755-3768.2009.01545.x

[70] Sadda SR, Campbell J, Dugel PU, Holekamp NM, Kiss S, Loewenstein A, et al. Relationship between duration and extent of oedema and visual acuity outcome with ranibizumab in diabetic macular oedema: a post hoc analysis of Protocol I data. Eye. 2020;34:480–90. 31320738 10.1038/s41433-019-0522-z PMC7042302

[71] Brown DM, Nguyen QD, Marcus DM, Boyer DS, Patel S, Feiner L, et al. Long-term outcomes of ranibizumab therapy for diabetic macular edema: the 36-month results from two phase III trials: RISE and RIDE. Ophthalmology. 2013;120:2013–22. 23706949 10.1016/j.ophtha.2013.02.034

[72] Gurung RL, FitzGerald LM, Liu E, McComish BJ, Kaidonis G, Ridge B, et al. Predictive factors for treatment outcomes with intravitreal anti-vascular endothelial growth factor injections in diabetic macular edema in clinical practice. Int J Retina Vitreous. 2023;9:23. 37016462 10.1186/s40942-023-00453-0 PMC10074667

[73] Diabetic Retinopathy Clinical Research Network, Wells JA, Glassman AR, Ayala AR, Jampol LM, Aiello LP, Antoszyk AN, et al. Aflibercept, bevacizumab, or ranibizumab for diabetic macular edema. N Engl J Med. 2015;372:1193–203. 25692915 10.1056/NEJMoa1414264 PMC4422053

[74] Veritti D, Sarao V, Soppelsa V, Lanzetta P. Managing diabetic macular edema in clinical practice: systematic review and meta-analysis of current strategies and treatment options. Clin Ophthalmol. 2021;15:375–85. 33551641 10.2147/OPTH.S236423 PMC7856351

[75] Chatziralli I. Editorial - Suboptimal response to intravitreal anti-VEGF treatment for patients with diabetic macular edema: is there any point in switching treatment? Eur Rev Med Pharmacol Sci. 2018;22:5047–50. 30070344 10.26355/eurrev_201808_15648

[76] Wykoff CC, Abreu F, Adamis AP, Basu K, Eichenbaum DA, Haskova Z, et al. YOSEMITE and RHINE Investigators. Efficacy, durability, and safety of intravitreal faricimab with extended dosing up to every 16 weeks in patients with diabetic macular oedema (YOSEMITE and RHINE): two randomised, double-masked, phase 3 trials. Lancet. 2022;399:741–55. 35085503 10.1016/S0140-6736(22)00018-6

[77] Wong TY, Haskova Z, Asik K, Baumal CR, Csaky KG, Eter N, et al. Faricimab Treat-and-extend for diabetic macular edema: two-year results from the randomized Phase 3 YOSEMITE and RHINE Trials. Ophthalmology. 2023;S0161-6420:00933–8. 10.1016/j.ophtha.2023.12.026. 10.1016/j.ophtha.2023.12.026 38158159

[78] Penha FM, Masud M, Khanani ZA, Thomas M, Fong RD, Smith K, et al. Review of real-world evidence of dual inhibition of VEGF-A and ANG-2 with faricimab in NAMD and DME. Int J Retina Vitreous. 2024;10:5. 10.1186/s40942-024-00525-9. 38233896 10.1186/s40942-024-00525-9 PMC10795384

[79] Wykoff CC, Garweg JG, Regillo C, Souied E, Wolf S, Dhoot DS, et al. KESTREL and KITE Phase 3 Studies: 100-Week Results With Brolucizumab in Patients With Diabetic Macular Edema. Am J Ophthalmol. 2024;260:70–83. 37460036 10.1016/j.ajo.2023.07.012

[80] Busch C, Zur D, Fraser-Bell S, Laíns I, Santos AR, Lupidi M, et al. Shall we stay, or shall we switch? Continued anti-VEGF therapy versus early switch to dexamethasone implant in refractory diabetic macular edema. Acta Diabetol. 2018;55:789–96. 29730822 10.1007/s00592-018-1151-x

[81] Kodjikian L, Bellocq D, Bandello F, Loewenstein A, Chakravarthy U, Koh A, et al. First-line treatment algorithm and guidelines in center-involving diabetic macular edema. Eur J Ophthalmol. 2019;29:573–84. 31238719 10.1177/1120672119857511

[82] Downey L, Acharya N, Devonport H, Gale R, Habib M, Manjunath V, et al. Treatment choices for diabetic macular oedema: a guideline for when to consider an intravitreal corticosteroid, including adaptations for the COVID-19 era. BMJ Open Ophthalmol. 2021;6:e000696. 34192155 10.1136/bmjophth-2020-000696 PMC8088120

[83] Sejournet L, Mathis T, Vermot-Desroches V, Serra R, Fenniri I, Denis P, et al. Efficacy and safety of fluocinolone acetonide implant in diabetic macular edema: practical guidelines from reference center. Pharmaceutics. 2024;16:1183. 39339219 10.3390/pharmaceutics16091183 PMC11435168

[84] Cicinelli MV, Cavalleri M, Querques L, Rabiolo A, Bandello F, Querques G. Early response to ranibizumab predictive of functional outcome after dexamethasone for unresponsive diabetic macular oedema. Br J Ophthalmol. 2017;101:1689–93. 28432109 10.1136/bjophthalmol-2017-310242

[85] Demir G, Ozkaya A, Yuksel E, Erdogan G, Tunc U, Celal Ocal M, et al. Early and Late Switch from Ranibizumab to an Intravitreal Dexamethasone Implant in Patients with Diabetic Macular Edema in the Event of a Poor Anatomical Response. Clin Drug Investig. 2020;40:119–28. 31768784 10.1007/s40261-019-00865-7

[86] Hernández Martínez A, Pereira Delgado E, Silva Silva G, Castellanos Mateos L, Lorente Pascual J, Lainez Villa J, et al. Early versus late switch: How long should we extend the anti-vascular endothelial growth factor therapy in unresponsive diabetic macular edema patients? Eur J Ophthalmol. 2020;30:1091–8. 31096782 10.1177/1120672119848257

[87] Ruiz-Medrano J, Rodríguez-Leor R, Almazán E, Lugo F, Casado-Lopez E, Arias L, et al. Results of dexamethasone intravitreal implant (Ozurdex) in diabetic macular edema patients: early versus late switch. Eur J Ophthalmol. 2021;31:1135–45. 32493065 10.1177/1120672120929960

[88] Malclès A, Dot C, Voirin N, Vié AL, Agard É, Bellocq D, et al. SAFETY OF INTRAVITREAL DEXAMETHASONE IMPLANT (OZURDEX): The SAFODEX study. Incidence and Risk Factors of Ocular Hypertension. Retina. 2017;37:1352–9. 27768641 10.1097/IAE.0000000000001369

[89] Rezkallah A, Kodjikian L, Malclès A, Dot C. DEX implant intravitreal injection, sustained intraocular hypertension, and steroid-induced glaucoma in patients with no risk factors. Graefes Arch Clin Exp Ophthalmol. 2018;256:219–20. 28801709 10.1007/s00417-017-3773-z

[90] Zarranz-Ventura J, Sala-Puigdollers A, Velazquez-Villoria D, Figueras-Roca M, Copete S, Distefano L, et al. Hospital Clínic—Hospital Vall de Hebron Intravitreal Dexamethasone Implant study group. Long-term probability of intraocular pressure elevation with the intravitreal dexamethasone implant in the real-world. PLoS ONE. 2019;14(Jan):e0209997. 30608950 10.1371/journal.pone.0209997 PMC6319768

[91] Sharma A, Kuppermann BD, Bandello F, Lanzetta P, Zur D, Park SW, et al. Intraocular pressure (IOP) after intravitreal dexamethasone implant (Ozurdex) amongst different geographic populations-GEODEX- IOP study. Eye. 2020;34:1063–8. 31570814 10.1038/s41433-019-0616-7 PMC7253432

[92] Ayaz Y, Erkan Pota Ç, Başol İ, Doğan ME, Türkoğlu Şen EB, et al. Anterior segment complications after dexamethasone implantations:real world data. Int Ophthalmol. 2023;43:4279–87. 37707746 10.1007/s10792-023-02838-4

[93] Levin AM, Chaya CJ, Kahook MY, Wirostko BM. Intraocular Pressure Elevation Following Intravitreal Anti-VEGF Injections: Short- and Long-term Considerations. J Glaucoma. 2021;30:1019–26. 34086610 10.1097/IJG.0000000000001894 PMC8635259

[94] Stoner AM, Lind JT, Gill Z, Seibold LK Anti-VEGF Injection IOP Elevations. EyeWiki®. American Academy of Ophthalmology. Updated July 24, 2022. Available in: https://eyewiki.aao.org/Anti-VEGF_Injection_IOP_Elevations#Long-term_sustained_IOP_elevation Last accessed May, 2024.

[95] Cicinelli MV, Rabiolo A, Capone L, Di Biase C, Lattanzio R, Bandello F. Factors associated with the response to fluocinolone acetonide 0.19 mg in diabetic macular oedema evaluated as the area-under-the-curve. Eye. 2023;37:242–8. 35094025 10.1038/s41433-021-01921-3 PMC9873740

[96] Goñi FJ, Barton K, Dias JA, Diestelhorst M, Garcia-Feijoo J, Hommer A, et al. Intravitreal Corticosteroid Implantation in Diabetic Macular Edema: Updated European Consensus Guidance on Monitoring and Managing Intraocular Pressure. Ophthalmol Ther. 2022;11:15–34. 34993882 10.1007/s40123-021-00427-1 PMC8770785

[97] Dot C, Poli M, Aptel F, Labbe A, Kodjikian L, Baillif S, et al. Ocular hypertension and intravitreal steroids injections, update in 2023. French guidelines of the French glaucoma society and the French ophthalmology society. J Fr Ophtalmol. 2023;46:e249–e256. 37302867 10.1016/j.jfo.2023.05.001

[98] Chang-Lin JE, Attar M, Acheampong AA, Robinson MR, Whitcup SM, Kuppermann BD, et al. Pharmacokinetics and pharmacodynamics of a sustained-release dexamethasone intravitreal implant. Invest Ophthalmol Vis Sci. 2011;52:80–86. 20702826 10.1167/iovs.10-5285

[99] Whitcup SM, Cidlowski JA, Csaky KG, Ambati J. Pharmacology of Corticosteroids for Diabetic Macular Edema. Invest Ophthalmol Vis Sci. 2018;59:1–12. 29297055 10.1167/iovs.17-22259 PMC5961100

[100] Authors nor listed. Comment traiter la migraine? [How to treat migraine?]. Available in: https://www.vidal.fr/maladies/douleurs-fievres/migraine/traitements-migraine.html Last accessed May, 2024.

[101] Ndubuisi Okonkwo O, Akanbi T, Thelma Agweye C Current Management of Diabetic Macular Edema [Internet]. Diabetic Eye Disease - From Therapeutic Pipeline to the Real World. IntechOpen; 2022. Available from: 10.5772/intechopen.100157 Last accessed May 4, 2024.

[102] Lai TYY, Lai RYK. Association between Retinal thickness variability and visual acuity outcome during maintenance therapy using intravitreal anti-vascular endothelial growth factor agents for neovascular age-related macular degeneration. J Pers Med. 2021;11:1024. 34683165 10.3390/jpm11101024 PMC8541068

[103] Sheth VS, Singer M, MacCumber M, Cutino A, Kasper J, Coughlin BA, et al. Long-term control of retinal thickness variability and vision following the 0.19mg Fluocinolone acetonide implant. J Vitreoretin Dis. 2023;7:490–7. 37974917 10.1177/24741264231201314 PMC10649457

[104] Kodjikian L, Bandello F, de Smet M, Dot C, Zarranz-Ventura J, Loewenstein A, et al. Fluocinolone acetonide implant in diabetic macular edema: International experts’ panel consensus guidelines and treatment algorithm. Eur J Ophthalmol. 2022;32:1890–9. 35139688 10.1177/11206721221080288

[105] Gonzalez VH, Luo C, Almeida DRP, Cutino A, Coughlin B, Kasper J, et al. Better baseline vision leads to better outcomes after the 0.19-mg fluocinolone acetonide intravitreal implant in diabetic macular edema. Retina. 2023;43:1301–7. 37130434 10.1097/IAE.0000000000003827

[106] Dobler E, Mohammed BR, Chavan R, Lip PL, Mitra A, Mushtaq B. Clinical efficacy and safety of intravitreal fluocinolone acetonide implant for the treatment of chronic diabetic macular oedema: five-year real-world results. Eye 2023;37:2310–5. 36513858 10.1038/s41433-022-02338-2 PMC9745701

[107] Rehak M, Busch C, Unterlauft JD, Jochmann C, Wiedemann P. Outcomes in diabetic macular edema switched directly or after a dexamethasone implant to a fluocinolone acetonide intravitreal implant following anti-VEGF treatment. Acta Diabetol. 2020;57:469–78. 31749051 10.1007/s00592-019-01439-x PMC7093402

