# Supplementary material for: What have we learned from a decade treating patients with diabetic macular oedema with 0.19 mg fluocinolone acetonide intravitreal implant?
Source: Eye (Lond). 2025 Feb 19;39(7):1238–48. doi: 10.1038/s41433-025-03692-7 (PMC12044149; doi:10.1038/s41433-025-03692-7)
Supplement: Supplementary file 1 — Supplementary Material [file 41433_2025_3692_MOESM1_ESM.docx]

**Supplementary Material**

Table S1. Overview of the functional and anatomic results of Fluocinolone intravitreal implants reported in randomized clinical trials.

| **Study** | **Ref** | **Duration (m)** | **Regimen** | **N (eyes)** | **BCVA (ETDRS letters)** | |  | **CRT (µm)** | |
| --- | --- | --- | --- | --- | --- | --- | --- | --- | --- |
|  |  |  |  |  | **Baseline** | **Change** | **≥15letters** | **Baseline** | **Change** |
| **FAME** | 20 | 24 | 0.2 µg/day FAc | 375 | 53.3 (12.7) | P=0.019 | 28.7ª^,**^ | 460.8 (160.0) | p≤0.003 |
|  |  |  | 0.5 µg/day FAc | 393 | 52.9 (12.2) | P=0.015 | 28.6ª^,**^ | 485.1 (173.8) | p≤0.003 |
|  |  |  | Sham | 185 | 54.7 (11.3) | -------- | 16.2 | 451.3 (152.0) | ------- |
| **FAME^‡‡^** | 21 | 36 | 0.2 µg/day FAc | 209 | N.P. | N.P. | 34 ª^,***^ | N.P. | N.P. |
|  |  |  | 0.5 µg/day FAc | 215 | N.P. | N.P. | 28.8ª^,**^ | N.P. | N.P. |
|  |  |  | Sham | 112 | N.P. | N.P. | 13.4 | N.P. | N.P. |
| **FAME^‡^** | 22 | 36 | 0.2 µg/day FAc | 165 | 54.7 (11.7) | 2.4 | 22.3^a^ | 466.6 (152.9) | -173.1^*^ |
|  |  |  | Sham | 72 | 55.7 (11.5) | 2.3 | 27.8^a^ | 435.0 (149.1) | -115.6 |
| **FAME(A)^‡^** | 22 | 36 | 0.2 µg/day FAc | 79 | N.P. | N.P. | 24.1^a^ | N.P. | N.P. |
|  |  |  | Sham | 35 | N.P. | N.P. | 28.6^a^ | N.P. | N.P. |
| **FAME(B)^‡^** | 22 | 36 | 0.2 µg/day FAc | 87 | N.P. | N.P. | 20.7^a^ | N.P. | N.P. |
|  |  |  | Sham | 37 | N.P. | N.P. | 27.0^a^ | N.P. | N.P. |
| **FAME^‡‡^** | 22 | 36 | 0.2 µg/day FAc | 209 | 52.2 (13.4) | 7.6^**^ | 34ª^,***^ | 456.2 (165.9) | -186.8 |
|  |  |  | Sham | 112 | 54.0 (11.5) | 1.8 | 13.3ª^,***^ | 461.8 (153.5) | -160.0 |
| **FAME(A) ^‡‡^** | 22 | 36 | 0.2 µg/day FAc | 110 | N.P. | N.P. | 31.8ª^,**^ | N.P. | N.P. |
|  |  |  | Sham | 59 | N.P. | N.P. | 13.6ª^,**^ | N.P. | N.P. |
| **FAME (B)^‡‡^** | 22 | 36 | 0.2 µg/day FAc | 99 | N.P. | N.P. | 36.4ª^,**^ | N.P. | N.P. |
|  |  |  | Sham | 53 | N.P. | N.P. | 13.2ª^,**^ | N.P. | N.P. |
|  |  |  | 0.5 µg/day FAc | 215 | N.P. | N.P. | 28.8ª^,**^ | N.P. | N.P. |
|  |  |  | Sham | 112 | N.P. | N.P. | 13.4 | N.P. | N.P. |

**Note:**

^*^ p<0.05 vs comparator/Sham.

^**^ *P*<0.01 vs comparator/Sham.

^***^ *P*<0.001 vs comparator/Sham.

^****^*P*<0.0001 vs comparator/Sham.

^a^Proportion of patients a ≥15-letter improvement in best corrected visual acuity (BCVA) from baseline at the year 3.

**FAME:** The specific data regarding BCVA and central retinal thickness are not available from the literature [20], hence are not listed in this table. The p value corresponded to the difference between fluocinolone acetonide intravitreal (FAc) implant and Sham.

^‡^ Nonchronic Diabetic macular oedema (DMO) (<3 Years)

^‡‡^ Chronic DME (≥ 3 Years).

**Abbreviations:**

Ref: Reference; m: Months; BCVA: Best-corrected visual acuity; EDTRS: Early Treatment of Diabetic Retinopathy Study; CRT: Central retina thickness; FAc: Fluocinolone acetonide intravitreal.

Table S2. Cataract and intraocular pressure related adverse events (AEs) in the FAME trials. Adapted from Campochiaro et al [21] and Cunha-Vaz et al [22]

| **FAME A + B [44]** | | | | |
| --- | --- | --- | --- | --- |
| **Phakic Patients, % (Study Eye)** | **Sham (n=121)** | **0.2 μg/day FAc**  **Implant (n=235)** | **0.5 μg/day FAc Implant (n=265)** | |
| Cataract related AEs  Cataract as AE  Cataract surgery | 50.4%  27.3% | 81.7%  80.0% | 88.7%  87.2% | |
| **Subjects, % (Study Eye)** | **Sham (n=185)** | **0.2 μg/day FAc**  **Implant (n=375)** | **0.5 μg/day FAc Implant (n=393)** | |
| IOP-related AEs  IOP elevation  Any IOP lowering medication*  ALT  Incisional glaucoma surgery | 11.9%  14.1%  0.0%  0.5% | 37.1%  38.4%  1.3%  4.8% | 45.5%  47.3%  2.5%  8.1% | |
| **FAME A + B [22]** | | | | |
| **Phakic Patients, % (Study Eye)** | **Nonchronic DME (<3 Years)** | | **Chronic DME (≥3 Years)** | |
|  | **Sham (n=54)** | **0.2 μg/day FAc**  **Implant (n=121)** | **Sham (n=66)** | **0.2 μg/day FAc**  **Implant (n=114)** |
| Cataract related AEs  Any Cataract  Cataract surgery | 26 (48.1%)  8 (14.8%) | 94 (77.7%)  91 (75.2%) | 34 (51.5%)  24 (36.4%) | 98 (86.0%)  97 (85.1%) |
| **Subjects, % (Study Eye)** | **Sham (n=72)** | **0.2 μg/day FAc**  **Implant (n=165)** | **Sham (n=112)** | **0.2 μg/day FAc**  **Implant (n=209)** |
| IOP-related AEs  IOP elevation as AE^†^  Any IOP lowering medication*  IOP related surgery^⁑^ | 6 (8.3%)  9 (12.5%)  1 (1.4%) | 67 (40.6%)  69 (41.8%)  7 (4.2%) | 16 (14.3%)  17 (15.2%)  0 (0.0%) | 72 (34.4%)  75 (35.9%)  11 (5.3%) |

*For a minimum of 7 days.

^†^Includes AE reports of ocular hypertension and IOP increased.

^⁑^Includes trabeculectomy, glaucoma surgery, or vitrectomy for elevated IOP.

Table S3. Cataract and intraocular pressure related adverse events (AEs) in the real-world studies.

| Study | Ref | Eyes (n) | LOFU, months | IOP related AES | Cataract Related AEs |
| --- | --- | --- | --- | --- | --- |
| **Alfaqawi et al.** | 23 | 28 | 12 | 3 (11%) | Not reported |
| **MEDISOFT** | 24 | 341 | 14 | 53 (15.4%) | Not reported |
| **Fusi-Rubiano et al** | 25 | 29 | 36 | 2 (6.9%) | Not reported |
| **USER** | 26 | 160 | 24 | 56 (35.0%) | Not reported |
| **Young et al** | 27 | 21 | 36 | 8 (38.1%) | Not reported |
| **Retro-IDEAL** | 28 | 81 | 36 | 27.2% | 21.3% |
| **IRISS** | 29 | 593 | 36 | 113 (19.1%) | Not reported |
| **PALADIN** | 30 | 115 | 24 | 27 (23.5%) | 8 (66.7%)* |
| **Panos et al** | 31 | 24 | 36 | 4 (16.7%) | Not reported |
| **Mushtaq et al** | 32 | 96 | 36 | 23 (24.0%)** | Not reported |
| **MEDISOFT** | 33 | 256 | 36 | 28.9%*** | Not reported |
| **Kodjikian et al**** | 34 | N.A. | 20^⁑^ | 20.1% | 31.47% |
| **REALFAc** | 35 | 62 | 24 | 17 (27.4%)^⁑^ | 2 (100%)^a^ |
| **PALADIN** | 36 | 94 | 36 | 26 (27.7%)*** | 18 (61.1%)^b^ |
| **Elbarky et al** | 37 | 22 | 12 | 5 (22.7%) | Not reported |
| **Fallico et al***** | 38 | 428 | 24  36 | 27% | 39% |
| **Khoramnia et al** | 39 | 695 | 24  36 | 236 (35.1%) | 33 (29.2%)^c^ |
| **Merrill et al** | 40 | 202 | 36 | 14 (18.2%)** | Not reported |
| **Ruiz-Moreno et al** | 42 | 31 | 24 | 9 (29.0%) | 5 (16.1%) |
| **Capone et al** | 46 | 241 | 24 | 14 (6%)^†^ | Not applicable^d^ |
| **Lebrize et al** | 47 | 221 | 13.4±12.5^e^ | 55 (24.8%) | 22 (10%) |

*Among the 12 phakic eyes.

**IOP ≥25 mmHg.

***IOP increase of ≥10 mmHg.

^†^IOP ≥30 mmHg

^⁑^Δ IOP 6–15 mmHg.

^a^Two phakic eyes were included.

^b^Among the 29 phakic eyes.

^c^Among the 113 phakic eyes.

^d^The study included only pseudophakic eyes.

^e^Range 2.4 to 33.5 months.

LOFU: Last observation follow-up


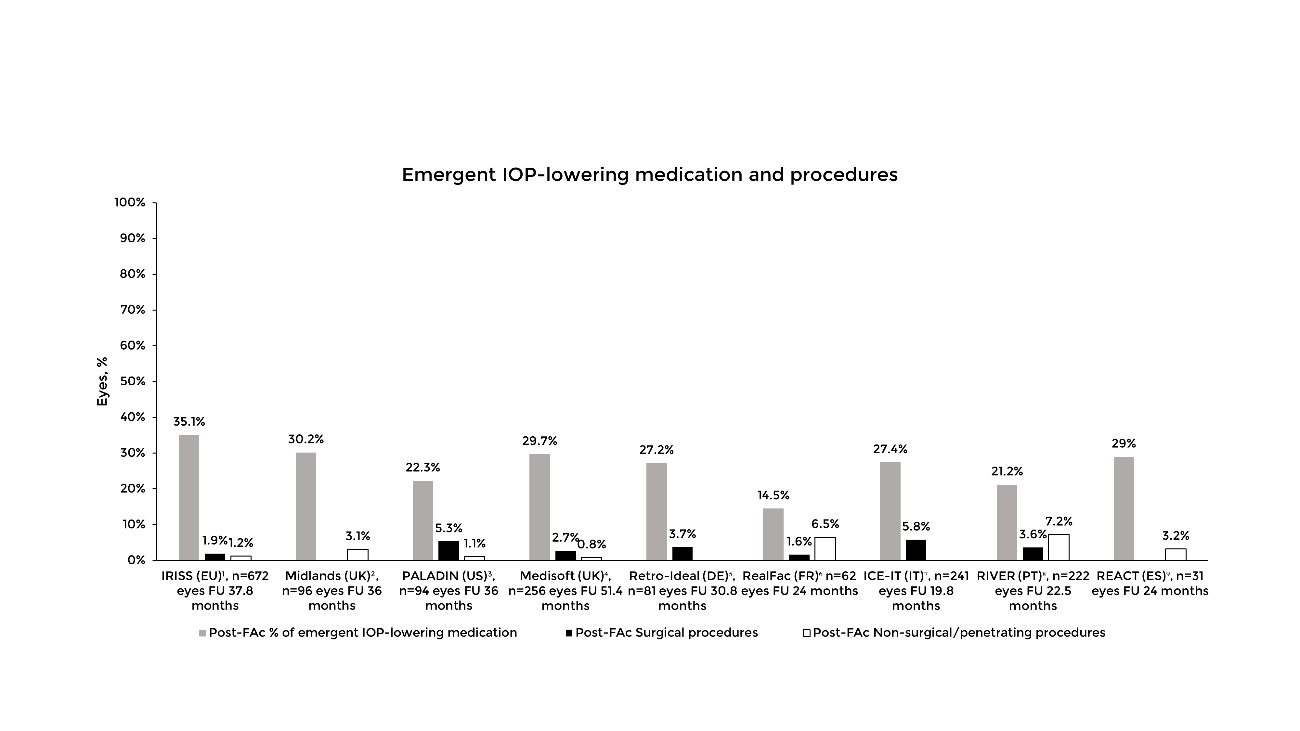


Figure S1. Overview of the proportion of emergent intraocular pressure (IOP)-lowering medications and surgical/non-surgical procedures in different real-world studies throughout their follow-up. Adapted from Augustin et al [28]; Mushtaq et al [32]; Bailey et al [33]; Mathis et al [35]; Singer et al [36]; Ruiz-Moreno et al [42]; Capone et al [47]; and Teixeira et al [49].

Studies: Midlands [Ref 32]; PALADIN [Ref 36]; Medisoft [Ref 33]; Retro-IDEAL [Ref 28]; RealFac [Ref 35]; ICE-IT[Ref 47]; RIVER [Ref 48]; REACT [Ref 42].

IOP: Intraocular pressure.


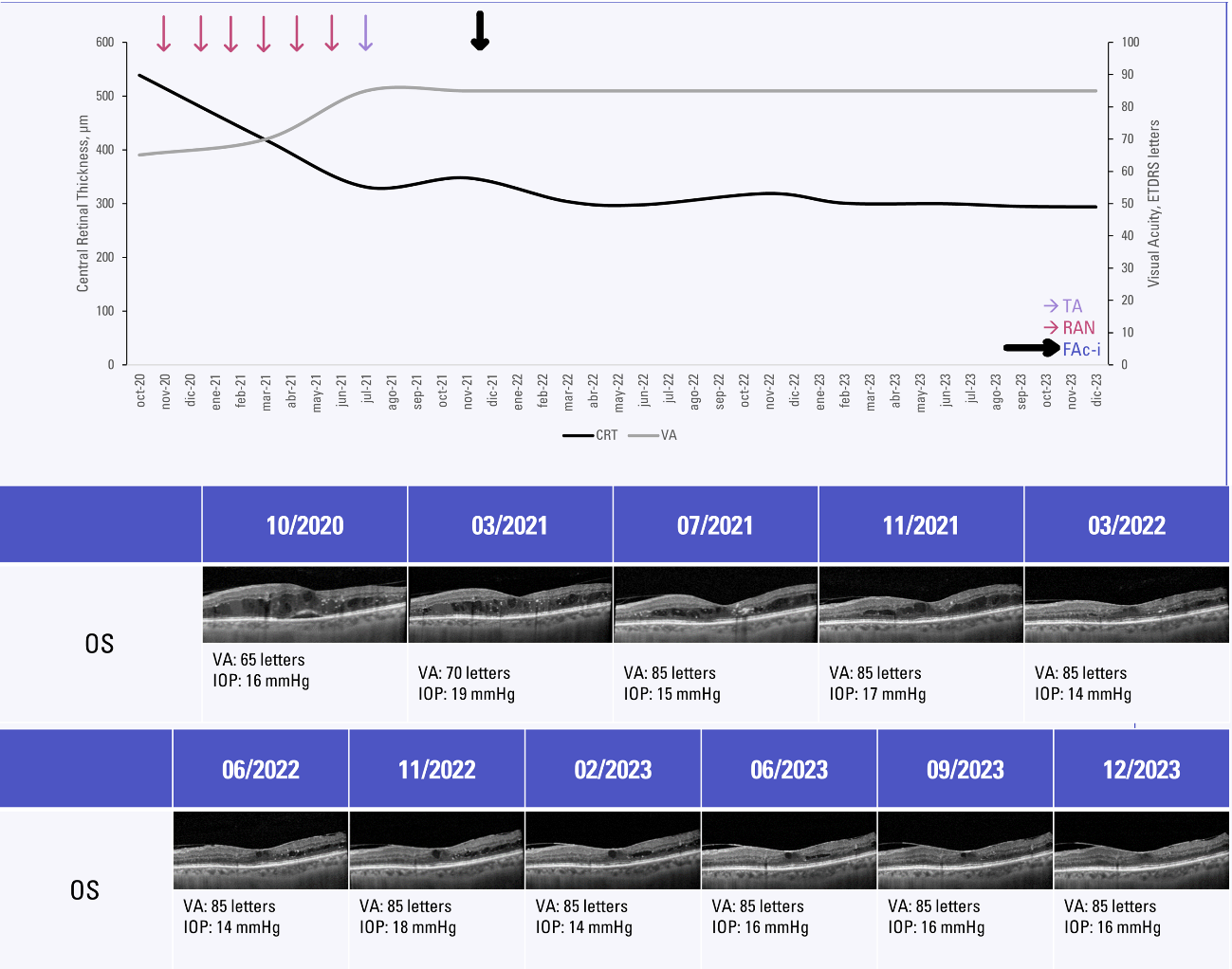


Figure S2. Case report of a 74 years-old patient who was diagnosed with moderate non-proliferative diabetic retinopathy with macular oedema in October 2020.

The patient started treatment with a vascular endothelial growth factor inhibitor (anti-VEGF) (Ranibizumab).

The left eye presented neurosensory detachment at baseline, and over time, there was a noticeable escalation from just a few hyperreflective foci at baseline to a gradual accumulation and merging of hard exudates near the fovea. Thus, the decision was made to start treatment with intravitreal steroids. The patient received treatment with a single intravitreal injection of a short-acting steroid intravitreal (triamcinolone acetonide). In December 2021 (see black arrow) the patient was treated with intravitreal fluocinolone acetonide (0.2 µg/day) implant.

After 2 years of FAc implantation, the eyes remain with stable VA and no oedema, with residual degenerative cysts and mild vitreo-retinal adhesion.

A. Overview of the central retinal thickness over the course of the study follow-up.

B. Optical coherence tomography images, visual acuity (VA) and intraocular pressure (IOP) throughout the study follow-up.

OS: Left eye.


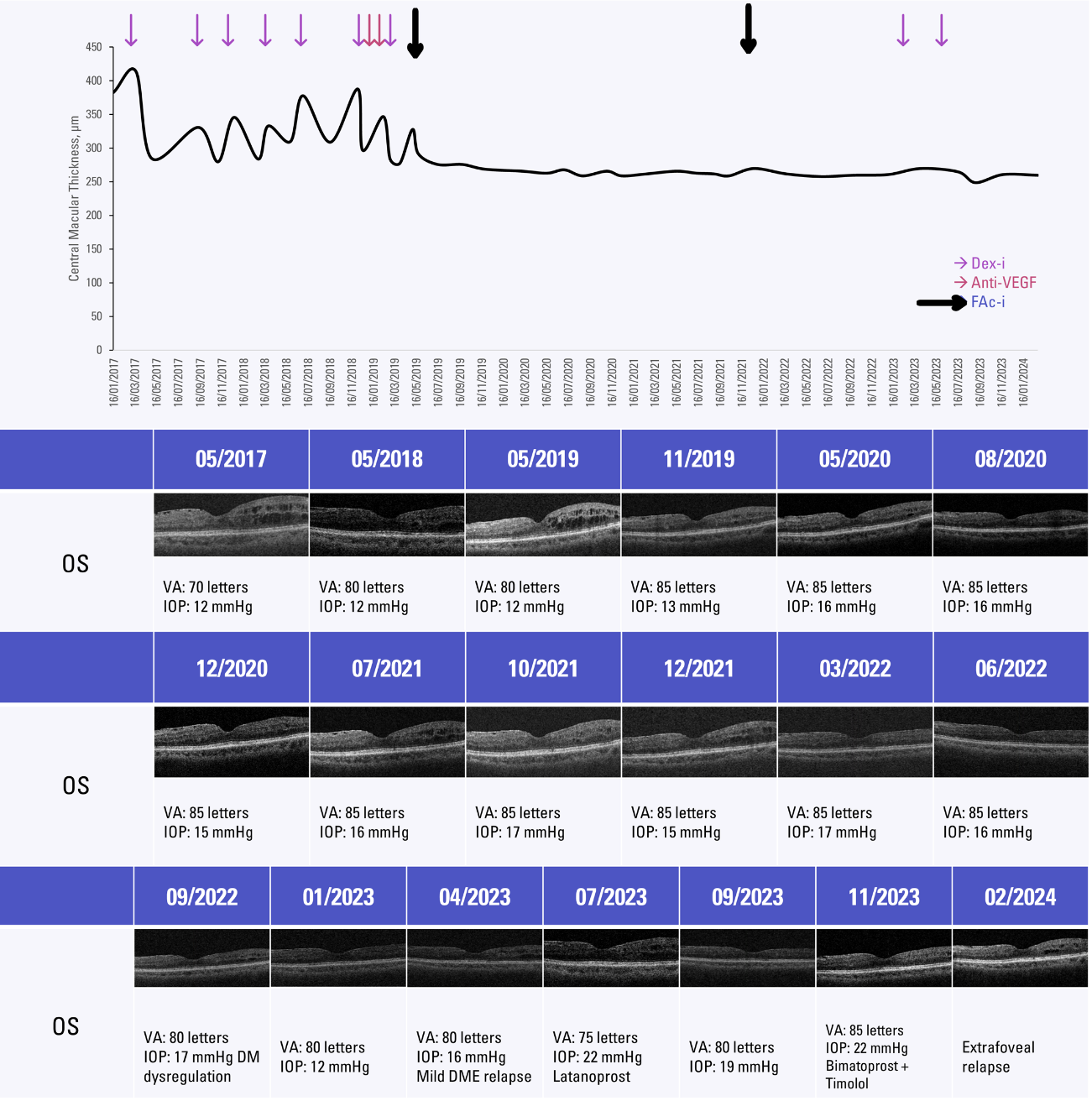


Figure S3. Case report of a 72 years-old patient with insulin dependent type 2 diabetes (diagnosed 30 years ago). HbA1c 7-8%. The patient was diagnosed with diabetic macular oedema in June 2107.
